# Using artificial intelligence (AI) to assess the prevalence of false or misleading health-related claims

**DOI:** 10.1098/rsos.240698

**Published:** 2024-10-02

**Authors:** Les Rose, Susan Bewley, Mandy Payne, David Colquhoun, Simon Perry

**Affiliations:** ^1^ Clinical Research Consultant, Retired, Salisbury, UK; ^2^ Emeritus Professor (honorary), Obstetrics & Women’s Health, Kings College London, c/o 10th floor St Thomas’ Hospital, Westminster Bridge Road, London SE1 7EH, UK; ^3^ Medical Editor (freelance), Pembroke Dock, Pembrokeshire, Wales, UK; ^4^ Emeritus Professor, Pharmacology, University College London, Gower Street, London WC1E 6BT, UK; ^5^ Independent Information Scientist, Oadby, Leicestershire, UK

**Keywords:** complementary healthcare, complementary medicine, complementary and alternative medicine, artificial intelligence, pseudoscience, misleading health claims

## Abstract

Complementary healthcare in the United Kingdom is subject to voluntary, publicly funded regulation. Many such practices include pseudoscience. There are concerns that regulated practitioners make misleading health claims. This study used an artificial intelligence (AI) tool to measure the prevalence of such claims. Websites operated by practitioners of pseudoscientific complementary and alternative medicine, registered with the Complementary and Natural Healthcare Council, were downloaded and assessed by the AI, which determined whether a website was relevant to the investigation and, if so, identified health-related claims that it judged as false or misleading, supplying a rationale. Of 6096 registrants, 1326 met the selection criteria, of which 872 clinics had 725 relevant and operational websites. The AI assessed text from 11 771 web pages, identifying false or misleading claims in 704 (97%) of the websites. The AI’s performance was quality-assured by four human assessors, who manually reviewed 23 relevant web pages. Humans identified on average 39.5 claims likely to be judged false or misleading by advertising regulators, the AI identified 36. Humans misidentified an average of 4.8 claims, AI misidentified two. Most practitioners of pseudoscientific therapies registered with the Complementary and Natural Healthcare Council make misleading health claims online. AI could support regulator efficiency.

## 1. Introduction

While considerable progress has been made in recent years to establish evidence as the basis for healthcare decisions, many practices are still based on poor science. A distinction can be made between practices which are based on plausible science but have weak clinical evidence, and those that are based on a system of theories, assumptions and methods erroneously regarded as scientific. The latter are termed pseudoscience [1].

In the consumer healthcare field, many practices are based on pseudoscientific claims. For example, reflexologists apply pressure to areas on the soles of the feet in accordance with their belief that there are physical connections between these and certain organs. Robust evidence does not support the existence of any such connections and nor does it support use of reflexology for any medical condition. Acupuncture is a component of traditional Chinese medicine in which thin needles are inserted into the skin at specific points believed to correspond to the affected regions of the body. Scientific investigation has not found any histological or physiological evidence for relations between these acupuncture points and the parts of the body they are claimed to treat and, despite a body of research that includes several thousand randomized controlled trials, there is no robust evidence of useful effects [2]. Nevertheless, these and many other practices are popular and provided by thousands of practitioners in the UK.

The growth of health-related pseudoscientific practices has resulted in demands for regulation [3,4]. In the UK, regulation of healthcare providers is by the Professional Standards Authority for Health and Social Care (PSA) [5], an independent body accountable to the UK Parliament. The PSA oversees 10 independent statutory healthcare regulators in the UK. Each publishes standards of competence and conduct expected of their profession, investigates complaints about members, checks quality of training courses and maintains a publicly accessible register of professional members. It is a criminal offence for anyone not on these registers to work in these regulated occupations. Regulated occupations include doctor, nurse, pharmacist and paramedic.

Two of these 10 statutory bodies regulate specific pseudoscientific practices: the General Chiropractic Council [6], established by the 1994 Chiropractors Act [7]; and the General Osteopathic Council [8], set up in 1997 following the Osteopaths Act [9]. As such, the terms ‘chiropractor’ and ‘osteopath’ are legally protected, and it is a criminal offence for an individual to describe themselves as either, unless they are registered with the appropriate regulator.

Membership of a PSA-regulated statutory healthcare body lends chiropractors and osteopaths credibility as bona fide healthcare practitioners, despite their practices being based on pseudoscience: chiropractic refers to the fictitious concepts of ‘innate intelligence’ and ‘vertebral subluxations’, which were conceived by the practice’s founder, Daniel David Palmer; the founder of osteopathy, Andrew Taylor Still, created the concept of ‘myofascial continuity’. None of these ideas has been validated by rigorous research.

For complementary and alternative therapists whose treatments are not covered by statutory regulators, a voluntary independent regulator also exists. The Complementary and Natural Healthcare Council (CNHC) was set up in 2008 with UK government support [10,11], for a range of therapies covered by the term ‘complementary medicine’. The CNHC defines ‘complementary’ as a treatment used *alongside* conventional medicine, and ‘alternative’ as treatment used *instead* of conventional medicine (M Coats 2024, personal communication), a distinction which is in line with that made by the US National Center for Complementary and Integrative Health [12]. In practice, the distinction is less clear, and they are often used interchangeably [13]. Given that the categorization is not related to the nature of the therapy, but to the *context* in which is it applied, individual complementary therapies may fall into either definition. Many of the practices are also pseudoscientific. Individuals who practise a complementary therapy may choose to register with the CNHC, but it is not mandatory nor regulated by law.

The CNHC is one of 32 medically affiliated bodies currently listed as Accredited Registers with the PSA, alongside groups as diverse as psychotherapists, hypnotherapists, cosmetic practitioners and counsellors. All such bodies are expected to meet PSA Standards for Accredited Registers. Therapists who choose to register with the CNHC benefit via the PSA’s Accredited Register programme [14]. They are permitted to display the PSA Quality Mark (logo). By virtue of their CNHC membership, and that organization’s accreditation with the PSA, a therapist’s CNHC registration lends credibility and generates trust in the eyes of the public. Furthermore, the UK government has reacted positively to proposals put forward by the Royal Institute for Public Health that members of Accredited Registers be considered part of the ‘wider public health workforce’ [15].

As well as meeting PSA Accredited Register standards, CNHC-registered practitioners are expected to ‘abide by our Code of Conduct, Ethics and Performance’ [16]. This Code instructs registrants to ‘use only factual and verifiable information when advertising’ their work or practice; registrants must not make unsubstantiated claims or mislead. It refers to the Code of Advertising Practice (CAP) published by the UK Advertising Standards Authority [17]. However, historical reports suggest that breaches of this instruction and of the CAP Code have been widespread. The PSA found that ‘approximately half [of CNHC registrants’ websites] appeared to deviate from the messaging that was developed by the CNHC and appeared to depart from the ASA’s guidance’; and ‘The Accreditation Panel agreed that the monitoring suggested that the issue of misleading advertising was potentially widespread amongst the CNHC’s registrant base’ [18].

Routine manual monitoring of members’ websites for compliance with their Code of Conduct is arduous. Given staff number limitations, the CNHC mainly relies on acting upon complaints raised by the public. Artificial intelligence (AI) presents an opportunity to automate the monitoring process, by scraping websites and scanning text for infringements, thereby enabling more thorough and consistent monitoring. An effective automated monitoring process would facilitate regulating bodies to identify practitioners whose websites violated their Code of Conduct, thus helping protect the public from exposure to misleading and unverifiable health claims.

We have not found evidence of other investigators using AI to monitor the marketing of pseudoscientific therapies. The Data Science team at the UK Advertising Standards Authority has developed an AI-based Active Ad Monitoring system [19], and it has been used to monitor environmental claims in airline advertisements [20]. Searches using strategies with variations of the following: (‘artificial intelligence’ OR AI) AND ‘regulatory compliance’ AND (medicine OR health OR therapy) AND (advertise OR website OR marketing) in the search engine Google Scholar did not retrieve any publications with a similar scope to this study.

As noted above, assessment of CNHC registrants has previously been attempted by the PSA [18]. While full details of the PSA’s methodology are not published, they describe a ‘spot check’ of websites of a random sample of 1% of registrants across all therapies. The PSA’s sample would have included websites promoting therapies that have an evidence base, such as sports massage and nutritional therapy, as well as those describing pseudoscientific therapies. By contrast, this study is concerned only with pseudoscientific therapies.

The purpose of this study is to use an AI tool to assess the extent of compliance with the CAP Code among CNHC registrants who practise pseudoscientific therapies.

### 1.1. Study objectives

1. To identify and quantify false or misleading claims made by practitioners of pseudoscientific therapies registered with the CNHC.
2. To analyse practitioners’ claims in relation to the evidence that exists for their specialism.

### 1.2. Expected outcomes of the study

It is hoped that the study results regarding compliance with the CAP as regards healthcare claims will inform the academic community, CNHC, other regulators and the general public about the extent of non-compliance in CNHC registrants’ websites. It will contribute to the body of evidence on the effectiveness of regulation for public protection and support the work of policymakers. Further, if an AI tool can be demonstrated to be cheap and effective for monitoring compliance with healthcare advertising regulations, it might be transferable to other regulators that aim to protect consumers from misleading health claims.

## 2. Material and methods

### 2.1. Glossary of terms

#### 2.1.1. Identified Claim

An *Identified Claim* is the term used here to refer to a quote or statement extracted by ChatGPT-4 when tasked with identifying potential false or misleading health claims on alternative therapy websites. This term specifically denotes the AI’s output, highlighting excerpts that may warrant further investigation. It does not presuppose the inaccuracy of the claim but rather points to what has been directly flagged by the AI without review.

#### 2.1.2. Hallucination

In the context of AI, ‘hallucination’ refers to a phenomenon where AI models, particularly in natural language processing and generative models, produce output that is not grounded in the input data or reality. This output can include fabricating facts, generating irrelevant or nonsensical information, or deviating significantly from the expected response based on the training data or the task at hand.

#### 2.1.3. Deserialization

Deserialization is the process of converting data encoded in a format suitable for storage or transmission back into its original structure or object. In the context of computing and data processing, serialization is the process of translating data structures or object states into a format that can be stored in a file, memory, or transmitted across a network. Conversely, deserialization takes this format and reconstructs the original data structure or object from it.

#### 2.1.4. JavaScript Object Notation

JavaScript Object Notation (JSON) is a lightweight data interchange format that is easy for humans to read and write, and easy for machines to parse and generate. JSON is text-based and language-independent, making it an ideal format for data interchange across various programming environments and systems. It is based on a subset of the JavaScript Programming Language Standard ECMA-262 3rd Edition, but its usage has expanded beyond JavaScript to become a common format in many programming languages for serializing and transmitting structured data over network connections.

### 2.2. Initial search of practitioners and websites

The CNHC was selected because it was set up with UK government support [10,11]. It is an Accredited Register with the Professional Standards Authority for Health and Social Care [21]. CNHC-registered members, who are permitted to practise without registration, instead make an active choice to sign up to a code of conduct that requires them to use only factual and verifiable information when advertising their work or practice, and not to make unsubstantiated claims or mislead.

A web scraping tool was used to download members from the CNHC website’s ‘find a practitioner’ tool at https://www.cnhc.org.uk/ [22]. The tool searched only for registrants based in the UK.

Websites were analysed for UK registrants who were registered with CNHC within any of the following therapy types: Alexander Technique, Aromatherapy, Bowen Therapy, Colon Hydrotherapy, Craniosacral Therapy, Healing, Kinesiology, Microsystems Acupuncture, Naturopathy, Reflexology, Reiki and Shiatsu.

For pragmatic reasons, including funding limitations, it was necessary to cap the number of websites that could be analysed. Thus, the following were excluded from the scope of the study: registrants who exclusively practised sports therapy, sports massage, massage therapy, hypnotherapy, yoga therapy or nutritional therapy. The rationale for excluding these registrants was that, although false or misleading claims may be made by practitioners of these therapies, the therapies themselves are not based on such pseudoscientific concepts. Registrants who practise both an included therapy type (e.g. Reiki) and a not specifically included therapy (e.g. sports massage) were included.

For each practitioner who had a website on their CNHC registry listing, the site was downloaded using a web crawler. The text was extracted from each page’s html. Text that was not integral to the page content, e.g. text in buttons and navigation menus, was removed from the content algorithmically.

Site downloads were then limited to 100 pages per website. The pages were found as follows: starting from the URL given on the practitioner’s CNHC registry listing, the web crawler followed the links on each page, eliminating those that go to other websites or that go higher in the folder structure than the original page. It continued to download pages and follow links until it had downloaded up to 100 pages from that website. Although the study plan was to limit the analysis to 30 pages per site, it was agreed to download 100 pages per site to leave open the opportunity to expand the number of pages for analysis subsequently, in the event of inconclusive findings.

Web pages not linked to via the publicly available pages could not be accessed with this system. Likewise, pages requiring log-in (e.g. to view patient records) were not retrieved, so that material remained confidential to the practitioner.

### 2.3. Data collection

The CNHC website was scraped over 2 hours between 14.15 and 16.15 on 23 November 2023. The web pages were downloaded over approximately 12 h between 16.46 on 23 November and 04.43 on 24 November 2023.

### 2.4. Data analysis

The main text from the first 30 pages from each website was then sent to OpenAI’s application programming interface (API) to be interrogated using their most advanced large language module (LLM), ChatGPT−4 (taking 9 days between 28 November and 7 December 2023). For reasons of cost and speed, the newer preview variant called gpt−4–1106-preview was used [23]. The prompt was as follows:

  Upon receiving the following piece of INPUT TEXT from an alternative medicine website:

  --- BEGIN INPUT TEXT ---

  {text}

  --- END INPUT TEXT ---Proceed as follows without additional commentary:

  1. Determine if the overall content is related to alternative medicine practices, therapies or claims.

  2. If the content is unrelated to alternative medicine, invoke the FlagAsIrrelevant() function.

  3. If the content is related, carefully review the text for any explicit or implied claims about the effectiveness of treatments in curing, treating or managing illnesses or diseases.

  4. Compile a list of EXACT quotations of any claims that are scientifically unsupported or have been refuted by established medical research. For each quotation, also prepare a brief explanation of why it is considered false or misleading.

  5. Once all quotations and their explanations have been compiled, call the ReportClaim() function, providing the list of quotations and corresponding reasoning.

  6. If there are no false or misleading claims to report, use the ReportNoFalseClaims() function to indicate that no such claims were found.

  Act on these directives succinctly.

  Proceed with the assessment according to these instructions.

The temperature parameter was set to 0 in the API to reduce randomness in the AI’s output. This parameter adjusts the creativity of responses: a low temperature gives predictable answers, while a high temperature introduces random variation to the response to give more varied and creative outputs. By setting the parameter to 0, ChatGPT should always respond to the same prompt with an identical answer.

Function Calling within OpenAI’s API makes it possible for ChatGPT to make calls to specific functions defined within the API call. We used function calling to request that the response from the AI was returned in a strictly formatted way that could be interpreted programmatically by defining two functions—ReportClaim and FlagAsIrrelevant.

The ReportClaim function requests parameters of exact text and the reasoning of why it was considered false or misleading for each quotation found in a clear format.

The FlagAsIrrelevant and ReportNoClaims functions take no arguments; however, the system would log when they have been called.

The data sent to ReportClaim were saved for later analysis.

The software stopped sending pages to the AI if, after assessing five pages, all five were flagged as irrelevant by the AI.

As it is relatively common for ChatGPT’s response to write the function call into the main content of the message rather than sending it as a separated function call as it should, some code was written to handle this and process the function call even though it was not called correctly.

### 2.5. Data quality assurance: human check on accuracy of AI

In view of the well-documented tendency of AI to confabulate (or ‘hallucinate’ [24]), it was considered important to have humans check its judgements. Four authors (S.B., L.R., M.P. and D.C.), working independently of each other, assessed a random sample of 30 of the Assessed Web Pages. The 30 pages were selected randomly by the software. The instructions they followed are shown in electronic supplemental material, Appendix 1 [25]. After the initial independent assessments, the four humans conferred in order to reach agreement on which pages were relevant, and which claims were false/misleading and to ‘verify’ the AI. First, the humans discussed and arrived at an agreement over which of the pages were relevant as compared with those judged relevant by the AI (Relevant Pages); then they discussed which of the claims identified, whether by AI or humans, were likely to be considered false/misleading by the UK advertising regulator. After elimination of claims which the authors considered not significantly misleading, the remaining claims were labelled as *Identified Claims* for the purposes of this study. The average number of *Identified Claims* made by the humans was calculated and compared with that of the AI. The author of the instructions (S.P.) did not participate in the assessment. Table 5 shows the results.

### 2.6. Inclusions and exclusions

It is possible that claims with respect to therapy types not regulated by CNHC could be made by other therapists at the same practice, and hence appear on the registrant’s website. These are included in our study because the registrant lists their website on the CNHC website, and therefore both registrant and CNHC are promoting them as part of the registrant’s marketing material.

Where websites were excluded from the analysis because the AI system was unable to find any relevant content we categorized (post hoc) why they were being flagged as irrelevant (table 2).

### 2.7. Data management and statistical analysis

Deserialization* problems were found when trying to interpret the JSON* data returned by ChatGPT. These were caused by two types of issue: (i) quotation marks which had not been escaped within strings (in JSON, text strings are stored within quotation marks, e.g. '........'. If there is a quotation mark within the string this can end the string. Hence, quotation marks are ‘escaped’ by placing a backslash before them to instruct the interpreter not to end the text string at that point. ChatGPT had sometimes missed this backslash); (ii) the use of curly quotation marks ‘…’ instead of straight quotation marks '…..' to enclose strings. These were edited manually after they were saved.

This is not a comparative study, so analytical statistics were not required. Descriptive statistics are expressed in simple frequencies derived from the AI tool output.

### 2.8. Primary outcome measure

The prevalence of AI-identified false or misleading claims (*Identified Claims*) made by practitioners registered with the CNHC for the provision of each therapy (listed above).

## 3. Results

A summary of the data extraction process and the finding of AI-identified false or misleading claims (*Identified Claims*) are shown in table 1. The complete list of claims identified by AI, with the reasons that AI provided, can be downloaded at [26] (an 8.1 Mb pdf file, with 2370 pages).

**Table 1.** Data extraction process and the finding of AI-identified false or misleading claims (Identified Claims).

group	number (or %)
CNHC registrants stated on CNHC website:	6217
CNHC registrants found during web scrape (Found Practitioners):	6096
Found Practitioners with relevant disciplines (Relevant Practitioners)^a^:	1326
clinics with which CNHC registrants were associated, found during web scrape (Found Clinics):	4055
Found Clinics who have at least one Relevant Practitioner (Relevant Clinics):	872
Relevant Clinics which list websites:	872
Relevant Clinics with valid websites URLs:	871
distinct, valid websites from Relevant Clinics (Distinct Websites):	864
Distinct Websites where at least one page was able to be downloaded (Operating Websites):	780
web pages (maximum 100) downloaded from Operating Websites (Downloaded Web Pages):	20 562
Downloaded Web Pages with malware detected:	5
Downloaded Web Pages without malware found (Web Pages):	20 557
Web Pages once limited to first 30 per website (Study Web Pages):	11 975
Study Web Pages not assessed by AI because first five pages on a website contained irrelevant content:	204
Study Web Pages assessed by AI (Assessed Web Pages):	11 771
Assessed Web Pages assessed by AI as having irrelevant content:	3226
Assessed Web Pages assessed by AI as having relevant content (Relevant Pages):	8545
Relevant Pages with no Identified Claims:	3006
Relevant Pages^b^ with Identified Claims:	5539
% of Relevant Pages with Identified Claims:	65%
total number of Identified Claims found:	23 307
average number of Identified Claims per Relevant Page:	2.7
Operating Websites that have relevant content (Relevant Websites):	725
average number of Identified Claims per Relevant Website:	32.1
number of Relevant Websites^a^ with Identified Claims:	704
proportion of Relevant Websites that have Identified Claims:	97%

^a^ Number of distinct operational websites evaluated (as some clinics list the same website).

^b^ Analysis limited to first 30 pages per website.

The AI assessed 11 771 web pages. Of 6217 registrants claimed by the CNHC website, 6096 UK registrants were able to be found using the web scraping tool. Of the registrants the tool found, 1326 met the study selection criteria. The number of websites found with respect to these registrants, that were operational and contained relevant content, was 725 (see table 1). Overall, the prevalence of *Identified Claims* made by practitioners registered with the CNHC to practise therapies in scope of this study was in 97% of registrant websites analysed. The range for individual therapies was 84–100% (as shown in table 2).

**Table 2.** Numbers of Identified Claims, by complementary therapy type, website and web page.

	# practitioners	# clinics	# downloaded websites	# downloaded web pages	# irrelevant pages	# relevant pages	# relevant pages without identified claims	# relevant pages with identified claims	# identified claims	# identified claims per page	# identified claims per downloaded website	% of relevant content websites with identified claims, n/N (%)
Alexander Technique teaching	71	55	47	1456	217	484	292	192	667	1.38	14.19	36/43 83.72%
aromatherapy	132	71	60	1910	324	632	240	392	1654	2.62	27.57	51/53 96.23%
Bowen therapy	59	46	39	946	150	443	126	317	1307	2.95	33.51	36/37 97.30%
colon hydrotherapy	61	48	40	1337	203	524	134	390	1640	3.13	41	37/37 100%
craniosacral therapy	45	36	32	757	109	275	122	153	612	2.23	19.12	28/29 96.55%
healing	46	25	21	423	68	221	93	128	556	2.52	26.48	20/20 100%
kinesiology	40	24	19	587	83	218	68	150	611	2.8	32.16	18/18 100%
microsystems acupuncture	42	38	32	1063	159	446	127	319	1401	3.14	43.78	28/29 96.55%
naturopathy	73	55	51	1598	182	627	187	440	2011	3.21	39.43	43/45 95.56%
reflexology	449	280	256	4507	905	2299	704	1595	6581	2.86	25.71	236/240 98.33%
Reiki	266	174	156	4979	682	2058	797	1261	5449	2.65	34.93	146/149 97.99%
Shiatsu	42	32	28	1003	147	319	116	203	820	2.57	29.29	26/26 100%

**Figure 1.**
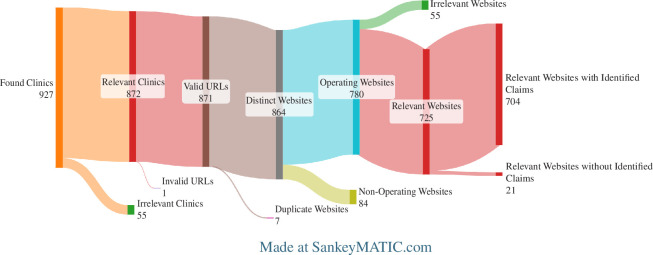
Sankey diagram showing attrition of numbers from Relevant Clinics to Relevant Websites with or without Identified Claims.

**Figure 2.**
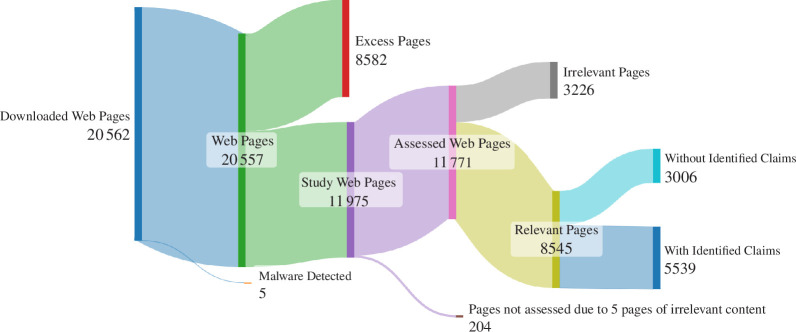
Sankey diagram showing attrition of numbers from Downloaded Web Pages to those with and without Identified Claims.

The number of relevant clinics found by the search was 872, of which 725 had operational, relevant websites. Of these, 704 had *Identified Claims* on their websites. Of 8545 relevant pages, 5539 contained *Identified Claims*. The data extraction and study results are displayed graphically in figures 1 and 2.

Ten instances of deserialization problems were found, as explained in the glossary.

The numbers and categorizations of exclusions are shown in table 3.

**Table 3.** Excluded websites (numbers and reasons).

reason	count	definition of reason
biographical notes of practitioner	1	as per reason
business closed message/empty site/under construction	6	The website is either a blank page or a simple web page announcing that the business is now closed, or the website is under construction.
content reading failure	12	as per reason
domain name	17	The website is no longer operational and is now a holding page for the hosting company trying to sell the domain name.
email address	8	An email address is listed instead of a website. The http:// was presumably added automatically by the CNHC’s website software, turning them into valid web addresses with embedded credentials, e.g. http://john.smith@gmail.com. This address simply redirects to the gmail.com website which of course contains no relevant information.
mostly irrelevant content	2	Tthis is where alternative medicine information makes up only a tiny portion of the content and so the AI has flagged it as irrelevant. One example was a page showing large numbers of beauty services with a very small section on alternative medicine.
not a practitioner website	9	The website was about another subject, sometimes the website of a hospice where the practitioner works. In some cases, it may have been the practitioner’s website, but they did not mention a relevant therapy.
total number of excluded websites	55	

In all 14 cases where the software had incorrectly identified a website as irrelevant (12 content reading failures and two where content was mostly irrelevant), the website contained at least one false or misleading claim.

Sixty-five per cent of relevant, eligible pages contained one or more *Identified Claims*, with a mean of 2.7 *Identified Claims* per page, and a mean number of *Identified Claims* per relevant website of 32 (s.d. 27.5, range 0–159). These data are further summarized in table 2.

Examples of AI output showing some website claims, with the rationale given by the AI for identifying the claim, are given in table 4.

**Table 4.** Examples of AI output.

claim on website	AI assessment
A chronic knee problem, which neither my GP or physiotherapist could improve, was resolved through acupuncture after three treatments.	The claim implies a definitive resolution of a chronic condition through acupuncture, which may not be supported by robust scientific evidence. The effectiveness of acupuncture for chronic knee problems is still a subject of ongoing research and debate.
Experience has allowed me to support myself, family, friends and clients with natural therapies to re-balance diseases ranging through anxiety, depression, muscular and joint pain, dermatitis, eczema, psoriasis, anorexia, weight loss, alcoholism, thyroid and adrenal disorders, infertility, insomnia, infant colic, weight loss and many others.	This claim suggests a broad and unspecific effectiveness of natural therapies for a wide range of medical conditions, which is not supported by sufficient scientific evidence. The term 're-balance diseases' is vague and not medically recognized.
Aqua Detox, a unique therapy based on the research of Dr Royal Rife, enables millions of ions to enter your body through the feet and begin to detoxify your body.	The claim that ions can enter the body through the feet and detoxify the body is scientifically unfounded. The concept of detoxification through the feet is not supported by credible scientific evidence.
The benefits of the Aqua Detox system are detoxification, stimulation, and balancing of the body’s energy meridians, and ATP production.	The claim that the Aqua Detox system can detoxify the body, balance energy meridians and stimulate ATP production is misleading. There is no scientific basis for the concept of energy meridians, and the body's ATP production is a complex biochemical process not influenced by such treatments.
Many conditions have improved, including reduction of pain and discomfort after chemotherapy, relief from allergic reactions, calming of eczema, low energy levels, arthritic pain in the joints and ionic detox also seems to work very well in curing hangovers.	This claim suggests that the Aqua Detox treatment can improve a variety of medical conditions, which is misleading. There is no scientific evidence to support the effectiveness of ionic detox treatments for these conditions.
research by Dr Chaudhury into the Aqua Detox System	The text implies that there is credible scientific research supporting the effectiveness of the Aqua Detox System. However, without access to the actual study and its peer-reviewed publication, this claim cannot be verified and is potentially misleading.
Over the past 20 years, I have blended prescriptive aromatherapy products to support individuals in the recovery and prevention of diseases including: eczema, nappy rash, pre-menstrual syndrome, muscular aches and pains, joint stiffness and pain, acne, dermatitis, colic, insomnia, anxiety, shingles, pneumonia, chicken pox, coughs and colds, sun protection, asthma, toothache, indigestion, psoriasis, circulatory problems and more.	This claim suggests that aromatherapy can support the recovery and prevention of a wide range of medical conditions. While aromatherapy may provide some benefits for relaxation and mood improvement, there is insufficient scientific evidence to support its effectiveness in treating or preventing the listed medical conditions. The claim may give consumers the false impression that aromatherapy is a substitute for conventional medical treatment.
When delivered transdermally through the skin the constituents of the oils are thought to be carried via the circulatory system to the muscle, organ or system of the body which is in need of support.	This claim implies that essential oils can target specific organs or systems in need of support when applied to the skin, which is a misleading oversimplification. The systemic absorption of essential oils through the skin and their therapeutic effects are not well-established in scientific literature, and the claim lacks evidence to support its specificity and efficacy.

### 3.1. Quality testing

The comparison of numbers of *Identified Claims* found by AI, with the numbers of those found by four human assessors, is shown in table 5.

**Table 5.** Comparison of false or misleading claims found by AI with human assessors (before and after verification process).

potential, or verified, false or misleading claims found	AI	human 1, S.B.	human 2, M.P.	human 3, L.R.	human 4, D.C.	average of humans
potential	36	56	43	30	48	44.3
verified	34	48	35	30	45	39.5

Of the 30 sampled pages, seven were found to be irrelevant. The 23 relevant pages were deemed to contain between 30 and 56 false or misleading claims by the four humans, and 36 were found by the AI. After conferring, verification and elimination of false positives, the average number of verified false or misleading claims identified by the humans was 39.5, compared with 34 identified by AI.

Despite all four humans identifying comparable numbers of false/misleading claims, they did not all identify the same claims. The average human assessor misidentified five quotations as false claims, whereas the AI only misidentified two.

## 4. Discussion

### 4.1. Principal findings

Ninety-seven per cent of websites of CNHC-registered practitioners of the 12 therapies considered pseudoscientific contained claims likely to be judged as false or misleading by an advertising regulator (‘*Identified Claims*’), in breach of the terms of their CNHC registration.

Claims likely to be judged false or misleading were present in 65% of analysed web pages, with an overall prevalence of 32 such claims per relevant website.

The performance of the AI tool used for this study was comparable to that of experienced human assessors in its ability to detect potentially false or misleading claims, and in its ability to justify its choices.

### 4.2. Strengths and limitations

Strengths include automation and quality assurance. Automation allows a whole dataset to be assessed and is thus more comprehensive and precise than an estimate based on a sample. It would be entirely impractical to conduct a study of this size manually with a human team. Websites are constantly evolving, so such a study could not be completed before the source material had changed substantially. Quality Assurance suggests that the AI is comparable to experienced human assessors in its ability to detect potentially false or misleading claims, and in its ability to justify its choices. The study found that AI and humans found comparable numbers of verified false/misleading claims in a sample of web pages. The AI reported very few false-negative claims, and both AI and humans were comparably lenient in their judgement of what was false/misleading. It is, therefore, likely that the total number of 23 307 *Identified Claims* found by AI in the 8545 relevant web pages of CNHC registrants is, if anything, an underestimate of the total number of false/misleading claims.

Limitations of this study include self-selection of practitioners, scope and replicability. It is possible that ‘dead’ or out-of-date ‘legacy’ websites might have been downloaded. They were, however, the ones listed on the CNHC website at the time of the downloads. A major methodological limitation is scope, as websites are not the only means to promote a practice and cannot reflect the face-to-face individual consultation. We were unable to assess channels such as social media and print advertising, nor could we assess verbal communications made during actual consultations, which might have a different rate of false or misleading claims. Practitioners might feel more constrained by written therapeutic claims on publicly accessible websites, and freer to make stronger claims verbally. Given that almost all practitioners made therapeutic claims on their websites, it is conceivable that the rate of advertising non-compliance found in our study could apply to the online presence of the pseudoscientific complementary healthcare sector in general.

The prompt sent to the AI used the term ‘alternative medicine’ rather than ‘complementary medicine’. Although theoretically this might have been expected to impact on the results, in practice, the AI still found that almost all registrants were advertising therapies that qualified under the selection criteria. This is because it is not possible for either the AI or humans to determine how the therapies are used.

It should not be assumed that the AI ‘reasons’ in the same way as a human. The results obtained are dependent on the prompt provided and the date of running the study as websites are constantly changing. A differently worded prompt could produce different results. For funding reasons, it was not possible to run the AI tool using several different prompts, and attempts were made to validate it by assessing a sample while blinded to the AI results. These factors affect replicability. The technology is still in development and cannot be expected to be precise. Sometimes, it constructs sentences that are not factually correct. However, the AI reported that almost all CNHC registrants are non-compliant, a result that was consistent with the sample of web pages assessed by humans. Any AI imprecision will have had minimal impact on the outcome of this study.

### 4.3. Interpretation

The prevalence of *Identified Claims* was high. Almost all CNHC registrants within the selection criteria and with relevant websites appear to mislead their clients to some extent by making claims in their websites that are not supported by evidence, which violates the terms of their registration with the regulating body the CNHC. The studied therapies were chosen because they were: (i) based on pseudoscientific concepts and (ii) lacked robust evidence of therapeutic effect. Therefore, practitioners would understandably have difficulty in promoting their businesses while simultaneously complying with their terms of registration. Nevertheless, their regulatory obligations remain.

Assuming that CNHC registrants are not deliberately misleading their clients, they must believe the claims they make. This would suggest that registrants use the word ‘evidence’ in a different way from that used in the evidence-based healthcare sector. This creates a double standard. Evidence in mainstream healthcare comes from basic science and methodology, including blinding, control groups and randomization to establish causality, whereas CNHC-registered practitioners may believe in less robust data, such as client testimonials, other anecdotes and unreliable ‘before-and-after’ case series.

### 4.4. Implications for policy, practice and research

The CNHC does not exist to promote complementary therapies; whether and how they work is for science to determine. The main aim of the CNHC is to protect the public. A key element of this regulatory protection with legal powers is to prevent them from being misled by online information in ways that might endanger their health or finances.

The regulator, and the Advertising Standards Authority, should address the finding that a large proportion of CNHC’s registrants routinely violate the Code of Conduct that is a condition of registration, through the information they use to market their practices online. Practitioner registration can be seen as an imprimatur of professionalism, which may be interpreted by potential clients as an ‘official’ or governmental endorsement of the therapies offered. If the regulator allows registrants to make potentially false or misleading claims, the CNHC will be seen to be supporting those false or misleading claims. It is difficult to see how practitioners of pseudoscientific therapies can advertise themselves as healthcare businesses that enhance health without violating advertising codes to be honest and truthful, and thereby misleading their clients. Thus, the ubiquity of the findings of this study calls the current effectiveness and rationale of regulation into question.

Training and support for registrants in how to avoid non-compliant advertising may lessen the numbers of violations, and concurrent monitoring by the regulator using AI would be a useful tool to support compliance. If implemented effectively, this could reduce levels of misleading online information by registrants and hence benefit their and the regulator’s image and authority. However, registrants may feel at a disadvantage by being constrained in the claims they can make online, and this could be a disincentive to CNHC registration. As the regulator is funded by registrant subscription (£70 per year), there is a conceivable conflict of interest were the regulator to discipline non-compliant registrants. Alternatively, registrants may simply move unevidenced claims offline, for example during a consultation.

While such training and monitoring may have only limited impact on the prevalence of poorly evidenced practices in the consumer health sector overall, it does have potential to give consumers a degree of protection by preventing their being misled by online information on the websites of members of an Accredited Register—a key aim of the government’s accreditation system. The software that was used in this study supports different versions of the websites over time, and in the future, it will be possible to observe the impact of any actions taken by CNHC.

While this study has demonstrated the potential for AI to speed monitoring of websites by regulators, the validation exercise and the resulting reflection on the kinds of discrepancies that arise between human and AI monitoring remind us of the need for human oversight. If AI can only be believed after it has been checked by humans, then its uses are limited. Its usefulness depends on building in human checks of the system to ensure it continues to perform effectively.

It is worthwhile acknowledging here an important limitation of AI as it exists currently. As the name, LLM, shows, AI is processing language, not knowledge. It works by assembling words in ways that simulate thought, which makes it appear to know more than it really does. These are early days for AI, which is why it was necessary to test its performance on a small sample of web pages. The outcome of this study, and the extent to which the AI findings align with those of the human assessors, make it clear that the tool’s limitations are less of a concern than they would be if we had found only half of registrants non-compliant, as the PSA reported in 2023 [18].

### 4.5. Future research

This work should be replicated in other settings and jurisdictions, and repeated following policy interventions to assess their impact. The AI tool could be applied to other healthcare regulators and groups of practitioners. Presently, much of the CNHC’s impact relies on their action taken in response to complaints submitted, after the event, by members of the general public. However, even some statutory regulators, such as the General Chiropractic Council, have been reluctant to discipline non-compliant registrants [27]. An AI tool used with appropriate safeguards (human checks as explained above) has the potential to improve practices prospectively if compliance becomes more easily measurable, especially if routine monitoring for compliance is made known to registrants, along with feedback, advice and warnings about any violations detected.

## 5. Conclusion

The overwhelming majority of practitioners registered with the CNHC who use pseudoscientific modalities are making false and/or misleading claims on their websites. This puts them in breach of their terms of registration.

An AI tool can be used to monitor websites of practitioners promoting pseudoscientific modalities for breaches of compliance with regulators’ codes of conduct for advertising, and does so with a level of accuracy comparable with that of human assessors. It presents an opportunity for regulators to offer more effective consumer protection from their members’ online misinformation than at present.

## Data Availability

The complete list of claims identified by AI, with the reasons that AI provided, can be downloaded at [26] (an 8.1 Mb pdf file, with 2370 pages). The web-scraping tool used to extract text from the websites in the study was created by S.P. for use monitoring and analysing health-related websites, particularly those associated with pseudoscientific health practices. It is available open source at [28]. The protocol for this study is published online: OSF | Protocol - AI project v2-0.pdf [29]. The instructions followed by human assessors for Data quality assurance: human check on accuracy of AI are in Appendix 1, available online: OSF | Rose et al_AI and Pseudoscience_RSOS-240698_Appendix.pdf [25].

## References

[1] Merriam-Webster dictionary . Definition ‘pseudoscience’. See https://www.merriam-webster.com/dictionary/pseudoscience (accessed 2 November 2023).

[2] Colquhoun D , Novella SP . 2013 Acupuncture is theatrical placebo. Anesth. Analg. **116** , 1360–1363. (10.1213/ANE.0b013e31828f2d5e) 23709076

[3] House of Lords . 2000 Features of an effective regulatory system. In Science and technology – sixth report. London: House of Lords Science and Technology Committee Publications. See https://publications.parliament.uk/pa/ld199900/ldselect/ldsctech/123/12307.htm#a29.

[4] Stone J , Matthews J . 1996 Complementary medicine and the law. Oxford, UK: Oxford University Press.

[5] Professional Standards Authority for Health and Social Care. See https://www.professionalstandards.org.uk/ (accessed 17 November 2023).

[6] General Chiropractic Council . What we do. See https://www.gcc-uk.org/about-us/what-we-do (accessed 17 November 2023).

[7] Chiropractors Act . 1994 London, UK: HMSO. See https://www.legislation.gov.uk/ukpga/1994/17 (accessed 17 November 2023).

[8] General Osteopathic Council . See https://www.osteopathy.org.uk/about-us/ (accessed 17 November 2023).

[9] Osteopaths Act . 1993 c. 21. London, UK: HMSO. See https://www.legislation.gov.uk/ukpga/1993/21 (accessed 17 November 2023).

[10] Complementary & Natural Healthcare Council . Who we are. See https://www.cnhc.org.uk/who-we-are (accessed 2 November 2023).

[11] The Prince’s Foundation for Integrated Health. Complementary therapists to be regulated by new complementary and natural healthcare council from April 2008. Archived press release. 7 January 2008. See https://web.archive.org/web/20080928145524/http://www.fih.org.uk/media_centre/natural_healthcare.html (accessed 10 October 2023).

[12] US Department of Health & Human Services . 2021 US National Center for Complementary and Integrative Health. Complementary, alternative, or integrative health: what’s in a name? See https://www.nccih.nih.gov/health/complementary-alternative-or-integrative-health-whats-in-a-name (accessed 17 November 2023).

[13] Bombardieri D , Easthope G . 2000 Convergence between orthodox and alternative medicine: a theoretical elaboration and empirical test. Health **4** , 479–494. (10.1177/136345930000400404)

[14] Professional Standards Authority . 2019 Our work with accredited registers. See https://www.professionalstandards.org.uk/what-we-do/accredited-registers (accessed 17 November 2023).

[15] Public Health England: the wider public health workforce: a review. See https://assets.publishing.service.gov.uk/media/5c7fc87fe5274a3f89509d74/The_wider_public_health_workforce.pdf (accessed 2 November 2023).

[16] Complementary & Natural Healthcare Council . Advertising your work or practice. In Code of conduct, ethics and performance, p. 22. Bristol, UK: The Complementary and Natural Healthcare Council. See https://www.cnhc.org.uk/uploads/asset/file/35/CodeofConductEthicsandPerformance.pdf.

[17] Advertising Standards Authority . See https://www.asa.org.uk/codes-and-rulings/advertising-codes/non-broadcast-code.html (accessed 2 November 2023).

[18] Professional Standards Authority for Health and Social Care . 2023 Decision on whether accreditation is in the public interest. Complementary and Natural Healthcare Council (CNHC). See https://www.professionalstandards.org.uk/docs/default-source/accredited-registers/panel-decisions/complementary-and-natural-healthcare-council-decision-on-whether-accreditation-is-in-the-public-interest-february-2023.pdf (accessed 2 November 2023).

[19] Advertising Standards Authority . 2023 AI-assisted collective ad regulation: the ASA’s 2024-2028 strategy. See https://www.asa.org.uk/static/42c50098-a985-4e98-858769e7218933c9/AI-assisted-collective-ad-regulation-ASA-Strategy-2024-2029.pdf (accessed 31 July 2024).

[20] Advertising Standards Authority . 2024 Clearing the air: using AI to monitor sustainability claims. See https://www.asa.org.uk/news/clearing-the-air-using-ai-to-monitor-sustainability-claims.html (accessed 31 July 2024).

[21] Professional Standards Authority for Health and Social Care . Find an accredited register. See https://www.professionalstandards.org.uk/what-we-do/accredited-registers/find-a-register (accessed 2 November 2023).

[22] Complementary & Natural Healthcare Council . Find a practitioner. See https://cnhc.org.uk/find-a-practitioner/ (accessed 31 July 2024).

[23] OpenAI . GPT-4. See https://openai.com/gpt-4 (accessed 2 November 2023).

[24] Ji Z *et al* . 2023 Survey of Hallucination in Natural Language Generation. ACM Comput. Surv. **55** , 1–38. (10.1145/3571730)

[25] Rose L , Bewley S , Payne M , Perry SA . 2024 Using artificial intelligence (AI) to assess the prevalence of false or misleading health-related claims made by practitioners of pseudoscientific therapies. (doi:10.17605/OSF.IO/Q95SA). Rose et al_AI and Pseudoscience_RSOS-240698_Appendix.pdf. See https://osf.io/gcsme. 10.1098/rsos.240698 PMC11523101 39479247

[26] Rose L , Bewley S , Payne M , Perry SA . 2024 Using artificial intelligence (AI) to assess the prevalence of false or misleading health-related claims made by practitioners of pseudoscientific therapies. (doi:10.17605/OSF.IO/Q95SA). claimsreport_10.17605OSF.IOQ95SA.pdf. See https://osf.io/hnuqs. 10.1098/rsos.240698 PMC11523101 39479247

[27] Rose L . 2020 Infantile chiropractic. See https://majikthyse.wordpress.com/2020/07/28/infantile-chiropractic/ (accessed 9 April 2024).

[28] perrys519 . 2024 Perrys519/shysterwatch: alpha release (v0.1.1). Zenodo. (https://zenodo.org/records/13254965)

[29] Rose L , Bewley S , Payne M , Perry SA . 2024 Using Artificial Intelligence (AI) to Assess the Prevalence of False or Misleading Health-Related Claims Made by Practitioners of Pseudoscientific Therapies. (doi:10.17605/OSF.IO/Q95SA). Protocol - AI project v2-0.pdf. See https://osf.io/9jvew. 10.1098/rsos.240698 PMC11523101 39479247

